# Plasma Copper and Zinc Levels in Ataxia–Telangiectasia

**DOI:** 10.3390/ijms27125315

**Published:** 2026-06-12

**Authors:** Annebelle E. H. Swinkels, Stefanie J. G. Veenhuis, Nienke J. H. van Os, Corry M. R. Weemaes, Nel Roeleveld, Michèl A. A. P. Willemsen

**Affiliations:** 1 Department of Pediatrics, Amalia Children’s Hospital, Radboud University Medical Center, 6525 GA Nijmegen, The Netherlands; stefanie.veenhuis@radboudumc.nl (S.J.G.V.); nienke.vanos@radboudumc.nl (N.J.H.v.O.); corry.weemaes@radboudumc.nl (C.M.R.W.); michel.willemsen@radboudumc.nl (M.A.A.P.W.); 2 Department of Pediatric Neurology, Amalia Children’s Hospital, Radboud University Medical Center, 6525 GA Nijmegen, The Netherlands; 3 Science Department IQ Health, Radboud University Medical Center, 6500 HB Nijmegen, The Netherlands; nel.roeleveld@radboudumc.nl

**Keywords:** Ataxia–Telangiectasia, copper, zinc, trace elements

## Abstract

Ataxia–Telangiectasia (A-T) is a rare neurodegenerative multisystem disease caused by mutations in the *A-T Mutated* (*ATM*) gene resulting in cerebellar ataxia, immunodeficiency and an increased cancer risk. Copper and zinc play important roles in similar cellular processes as the ATM protein, such as cell growth, apoptosis, and oxidative stress. This study aimed to explore copper and zinc levels in individuals with A-T since imbalances in these trace elements may contribute to the clinical features commonly seen in A-T and therefore be a target for novel therapies; furthermore we aimed to assess the potential role of copper and zinc as disease biomarkers. In this retrospective cohort study, plasma copper and zinc levels were collected from 37 individuals with A-T and compared with age-related reference values. The results showed lower zinc levels in pediatric individuals with classic A-T, but no differences in copper levels. In adults, copper levels were lower in classic A-T, but not in variant A-T. These findings suggest that copper and zinc metabolisms are dysregulated in A-T, but since mixed model analysis showed minimal changes over time, copper and zinc do not appear to be reliable biomarkers for disease progression.

## 1. Introduction

Ataxia–Telangiectasia (A-T) is a rare, neurodegenerative multisystem disease resulting from pathogenic variants in the *A-T Mutated* (*ATM*) gene, encoding the ATM kinase enzyme. This enzyme plays an essential role in various cellular processes, such as cell cycle control, DNA repair, apoptosis, oxidative stress management, and mitochondrial energy metabolism [1]. Classic A-T is characterized by the absence of ATM kinase activity, resulting in the onset of cerebellar ataxia during childhood, followed by extrapyramidal movement disorders, peripheral neuropathy, oculomotor apraxia, and oculocutaneous telangiectasias. Additionally, individuals with A-T suffer from both humoral and cellular immunodeficiency, progressive respiratory failure, and an increased risk of developing malignancies and diabetes [2]. No cure exists for A-T and individuals with classic A-T generally die before the age of thirty years due to respiratory insufficiency or malignancies [3]. Some individuals present with relatively milder phenotypes, designated as “variant A-T”, due to some residual ATM kinase activity [4].

There is a critical need to gain more insight into the pathophysiology of A-T, the identification of biomarkers for disease progression and their use as outcome measures in trials, and for new therapeutic interventions. This necessity is also described in a recent systematic review on biomarkers in A-T [5]. Trace elements are essential for brain health, as they regulate cellular energy, support structural protein stability, and counteract oxidative stress. Despite growing evidence for the pathophysiological involvement of essential trace elements, including copper and zinc, in neurodegenerative diseases such as Alzheimer’s and Parkinson’s disease [6,7] and reported alterations in copper and zinc levels in individuals with A-T [8,9], these trace elements are not included among the potential biomarkers discussed in the paper by Tiet et al. [5].

Zinc is one of the most crucial metals, with a key function not only in the structure and function of numerous proteins but also in cell growth, proliferation, and apoptosis. Moreover, zinc is essential for the immune system and serves as a protective agent against cell damage by oxidative stress from free radicals [10]. Furthermore, emerging evidence suggests a potential association between low blood zinc levels and carcinogenesis [11] as well as diabetes [10]. Copper is another essential micronutrient involved in several metabolic processes including oxidative phosphorylation, elimination of free radicals, iron metabolism, and synthesis of hormones and neurotransmitters. The role of copper in the development of chronic diseases is receiving increasing attention. Recent studies have reported elevated copper levels in various cancerous diseases [12]. Notably, Qin et al. [13] described a relationship between ATM and copper metabolism, reporting that ATM may play an important role in the regulation of intracellular copper concentrations.

The clinical symptoms in individuals with A-T and the pathophysiological functions of copper and zinc seem to concern similar processes. In addition, copper and zinc influence processes regulated by the ATM protein, such as aging, oxidative stress, and apoptosis. These findings underscore the need to investigate the levels of these metals in persons with A-T. Previous research reported increased copper and decreased zinc levels in a series of 16 individuals with A-T (aged 3–23 years) [8]. The only other study addressing this topic reported conflicting results, as zinc levels in individuals with A-T (*n* = 14, aged 3–20 years) were found to be similar to those in healthy controls. Copper levels were not studied [9].

The objective of the current study is to further investigate the copper and zinc plasma levels, and their association, in individuals with A-T to determine whether these metals could serve as biomarkers for monitoring disease progression or as potential therapeutic targets.

## 2. Results

A total of 37 individuals with A-T were included in this study, 22 males and 15 females. Of these individuals, 30 had classic A-T, with a median age of 9 years (range: 1–38 years), while 7 had variant A-T, with a median age of 28 years (range: 16–54 years) at the moment the first blood sample was taken. Retrospective data collection, covering the period from January 2019 through March 2024, yielded a total of 130 measurements of plasma copper and zinc levels over time with a median number of four samples per person (range 1–6). For each person with multiple measurements of copper and zinc, individual median values were used in the further analyses of population medians and ranges.

### 2.1. Pediatric Population

**Figure 1:**
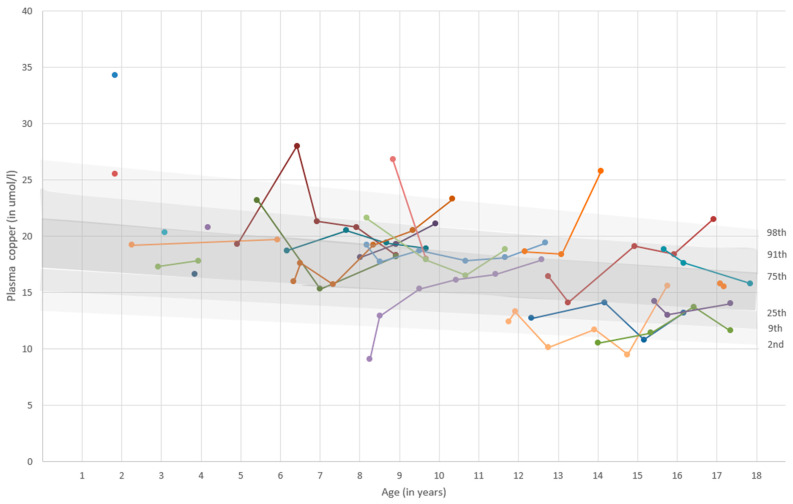
Plasma copper levels (in µmol/L) in children with classic A-T. Colored lines represent individual patient copper trajectories. Reference values (Al Fify et al. [14]) are shown as gray areas, with different shades corresponding to different percentiles.

**Figure 2:**
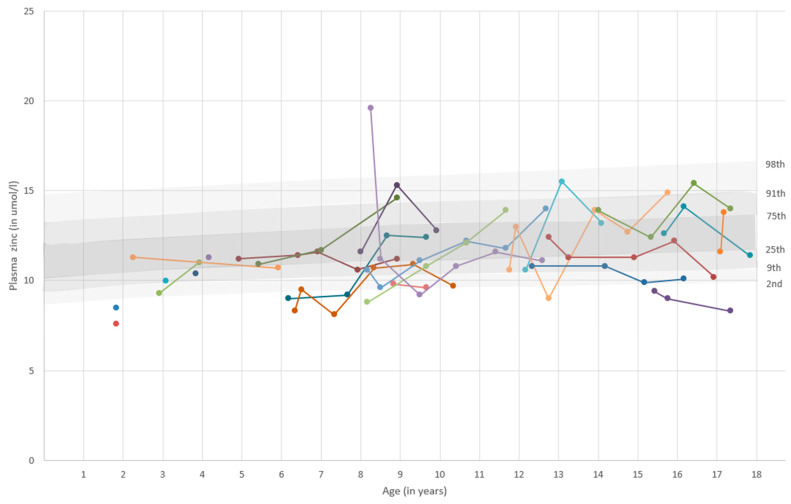
Plasma zinc levels (in µmol/L) in children with classic A-T. Colored lines represent individual patient zinc trajectories. Reference values (Al Fify et al. [14]) are shown as gray areas, with different shades corresponding to different percentiles.

A total of 81 measurements of copper and zinc were collected from 25 children (range: 1–17 years). Since one child only had variant A-T (with three measurements of copper and zinc), this individual was excluded from further analysis. No statistically significant differences in plasma zinc or copper levels were found between boys and girls with A-T. In children with classic A-T, the median plasma copper level was 18.4 µmol/L (range: 11.5–34.3 µmol/L), which did not differ from the reference values (*p* = 0.297) (see Table 1a and Figure 1). Zinc levels were statistically significantly reduced in children with classic A-T (median zinc level: 11.2 µmol/L, range: 7.6–14.0 µmol/L) as compared to reference values (*p* = 0.005) (Figure 2). This resulted in a higher copper/zinc ratio in children with classic A-T (median: 1.6, range: 0.9–4.0) as compared to age-related reference values (*p* = 0.011). Further subgroup analysis revealed that these differences in zinc levels and copper/zinc ratio were observed in younger children (aged 1–11 years) only and not in adolescents (aged 12–17 years), as depicted in Table 1b,c.

Using a mixed model analysis with individual measurements over time, an average decrease in plasma copper levels of 0.3 µmol/L (95% CI −0.58–−0.06) per year and an average increase in plasma zinc levels of 0.2 µmol/L (95% CI 0.04–0.29) per year were observed, in accordance with the slight age-dependent decrease and increase seen in the reference values.

### 2.2. Adult Population

A total of 49 measurements of copper and zinc were collected from fourteen adults aged 18–57 years, eight with classic A-T and six with variant A-T. Although sex-specific differences in copper and zinc levels were described in the literature [15], these differences were minimal in our reference cohort. Therefore, given the small number of adult individuals with A-T, male and female individuals were combined in the analyses.

As shown in Table 2a, 29 plasma copper levels in eight adults with classic A-T were available. The median copper level was 14.8 µmol/L (range: 12.1–18.1 µmol/L), which was statistically significantly lower compared to the reference values (*p* = 0.003). The median zinc level was 12.0 µmol/L (range: 9.7–14.3 µmol/L), and did not deviate from the control group (*p* = 0.592). The copper/zinc ratio in individuals with classic A-T was lower compared to the controls, although this difference did not reach statistical significance (median ratio: 1.3, range: 0.8–1.6) (*p* = 0.076).

In six adults with variant A-T (total of 20 measurements), the median plasma copper level was 18.0 µmol/L (range: 17.0–21.8 µmol/L), which was similar to the reference values (*p* = 0.739), as can be seen in Table 2b. Zinc levels were lower in adults with variant A-T, with a median of 10.7 µmol/L (range: 9.6–14.1 µmol/L), although this difference was not statistical significant (*p* = 0.088). In this group, the copper/zinc ratio was not statistically significantly different compared to the reference values (median 1.7 µmol/L, range 1.2–2.2). Using a mixed model analysis with individual measurements over time, an average increase in plasma copper levels of 0.1 µmol/L (95% CI −0.03–0.2) and an average decrease in plasma zinc levels of 0.1 µmol/L (95% CI −1.2–0.02) per year were observed in adults with classic or variant A-T.

## 3. Discussion

This study describes the presence of some deviating plasma levels of copper and zinc in patients with A-T. To our knowledge, this is the largest retrospective cohort study to date examining copper and zinc levels in pediatric and adult individuals with classic and variant A-T. Although the mixed model analyses demonstrated statistically significant changes in copper and zinc levels over time in the pediatric population, the magnitude of these changes was minimal and therefore unlikely to be clinically relevant, given the slight age-dependent changes in the reference values as well. Consequently, copper and zinc do not appear to be suitable biomarkers for disease progression. Nevertheless, other findings from this study offer valuable insights.

Squadrone et al. [8] reported elevated copper and decreased zinc levels in a series of 16 individuals with A-T between 3 and 23 years of age. Our study corroborates the finding of reduced zinc levels, particularly in children aged 1–11 years. Interestingly, we found that copper levels were statistically significantly lower in our classic A-T population starting from the age of 18 years, which is in contrast to Squadrone et al. This difference could be explained by the fact that Squadrone et al. did not stratify their analysis for the classic and variant A-T groups, whereas we found lower copper levels in adults with classic A-T only. As reported in the Introduction, copper and zinc were not discussed as potential biomarkers in the recent systematic review by Tiet and colleagues on biomarkers in A-T [5]. It should be noted that the study by Squadrone et al. met the inclusion criteria and was included in the review; for unclear reasons, however, copper and zinc were not addressed in the main text.

A-T is a multisystem disorder affecting various organs, including the brain, lungs, liver, and immune and endocrine systems. Additionally, “accelerated aging” is a clinically recognizable mechanism in A-T, and these individuals are at a markedly increased risk of developing cancer. Zinc plays a vital physiological role in nearly all the abovementioned organs and processes, such as cell growth, proliferation, and apoptosis. A zinc deficiency may, at least theoretically, contribute to disease severity or progression in several of these areas. While severe zinc deficiency typically presents as dermatitis, symptoms of mild to moderate deficiency can vary widely, ranging from cell-mediated immune dysfunctions to hair loss, decreased nerve conduction, ataxia, growth retardation, mental lethargy, or loss of appetite [16,17,18]. Although the median zinc level in our pediatric A-T cohort was above the clinical threshold set for “zinc deficiency” (9.95 µmol/L) [19], it remained statistically significantly lower compared to the healthy population, especially in children below 12 years of age.

Zinc deficiency can be either inherited, as seen in acrodermatitis enteropathica, or acquired due to inadequate intake, chronic illnesses such as diabetes, liver or kidney disease, chronic infections, or malabsorption issues linked to conditions such as chronic inflammatory bowel disease or pancreatic insufficiency [18,19]. Although progressive liver disease and diabetes mellitus are common in A-T, these conditions usually arise after the first decade of life [20,21]. Since we also observed lower zinc levels in children with A-T in their first decade of life, the underlying cause of low zinc levels in A-T cannot be a result of these endocrine or liver problems, and indicates that the underlying cause of low zinc levels from early childhood onwards in A-T necessitates further investigation.

Regardless of the cause, numerous studies have demonstrated that low blood zinc levels are associated with an increased risk of poor growth and retarded development, impaired innate and adaptive immunity, diabetes mellitus, and various malignancies [11,16]. Additionally, impaired zinc homeostasis may play a critical role in neurological conditions, such as Alzheimer’s disease and ataxia, as well as in ischemic stroke and traumatic brain injury [16,17,22]. Adequate zinc supplementation has been shown to mitigate some of these risks. In children, sufficient zinc supplementation has been linked to increased CD4^+^ and CD8^+^ cell counts [23] and reduces the incidence of lower respiratory tract infections [24], while in adults, it shortens the duration of viral respiratory infections [23]. Moreover, zinc supplementation in adults is associated with reduced levels of specific markers for oxidative stress and inflammation [25]. Evidence also suggests that zinc may exert anticancer effects by promoting apoptosis and reducing angiogenesis [26]. In diabetes, zinc supplementation has been shown to protect against the development of diabetic retinopathy [27] and to lower blood glucose levels in animal models [28].

Given the overlap between symptoms in patients with low zinc levels and patients with A-T, the potential benefits of zinc supplementation highlight the importance of considering additional zinc intake, even in the absence of evident deficiency, especially since adequate zinc supplementation is generally recognized as having minimal side effects [29,30]. Excessive intake can lead to acute toxicity, characterized by symptoms such as headache, nausea, gastrointestinal complaints, and, in rare cases, neurological symptoms, or to chronic toxicity, manifesting as copper deficiency caused by reduced intestinal absorption [16,24,31]. Due to this zinc-induced, impaired intestinal copper absorption, a trial of zinc supplementation may be especially relevant for individuals with elevated copper levels, but our findings also suggest that copper levels may spontaneously decrease over time (*n* = 2). On the other hand, this reduced copper absorption may be a critical concern in individuals with lower plasma zinc and plasma copper levels.

Both copper deficiency and toxicity are not favorable in individuals with A-T. A copper deficiency can adversely affect the immune system and oxidant defense system, leading to excessive oxidative stress, which is also a consequence of copper toxicity along with potential DNA damage and neurological symptoms such as ataxia [32]. Similar to copper deficiency, copper toxicity leads to oxidative stress, gastrointestinal complaints and headache; in addition severe copper toxicity may result in liver and kidney failure.

Besides copper and zinc levels, we investigated the copper/zinc ratios since previous research showed elevated ratios in children and adolescents with acute and chronic conditions, such as infections, chronic inflammation, type 1 diabetes mellitus, and metabolic syndrome [33]. In adults, increased serum copper/zinc ratios are associated with various malignant diseases [34,35]. We found slightly elevated ratios in children aged 1–11 years and in adults with variant A-T. These differences could possibly be explained by the higher prevalence of infections, diabetes mellitus, and malignancies within the A-T population compared with the general population [36]. Further research is necessary to investigate this association and to explain why this difference is not observed in all individuals with A-T.

This study has some limitations. Although it involves a relatively large A-T cohort—the largest A-T cohort in whom serum copper and zinc levels were studied to date—the numbers remain small due to the rarity of the disease and the necessity to use individual median values instead of all measurements in most comparisons to guarantee independence. Another limitation of this study is that the reference cohorts and the Dutch A-T cohort may have differed in demographic characteristics and laboratory procedures, which may have affected the comparability of the results. Additionally, we did not account for well-known factors that may influence copper and zinc levels, such as infection, low (meat) intake and liver disease [18], which might both be common in individuals with A-T. In addition, this study did not account for the potential use of oral contraceptives or dietary supplements containing copper or zinc.

In conclusion, this study revealed lower serum zinc levels, particularly in young children with classic A-T, although these levels remained above the threshold for an evident deficiency. Given the overlap in symptoms seen in individuals with A-T and in individuals with zinc deficiency, zinc supplementation may be tested in future interventional studies as a rational therapeutic approach. Further studies are needed to assess whether zinc supplementation leads to clinical improvements in individuals with A-T.

## 4. Materials and Methods

This retrospective, single-center study was conducted among individuals with a genetically confirmed diagnosis of A-T who were followed at the multidisciplinary outpatient clinic of the Radboud University Medical Center in Nijmegen, the Netherlands. As part of the annual check-ups, routine blood tests were performed, including measurements of plasma copper and zinc levels. Data on date of birth, gender, A-T subtype, and copper and zinc levels were retrieved from the medical records. All individuals with at least one measurement of copper and/or zinc were included in this study.

Age-dependent reference values for plasma copper and zinc are well established for both the pediatric population (i.e., 0–18 years) [14,37] and adults [38]. For this study, we used plasma copper and zinc reference values from Al Fify et al. [14] for A-T individuals up to the age of seventeen years.

In adults, intervals of reference values are gender dependent [15,38]. Similar to the study by Andrew et al. [15], plasma levels of the adult A-T population were compared to reference values derived from the large analytical database compiled in the US National Health and Nutrition Examination Surveys (NHANES) [39], conducted by the US Centers for Disease Control and Prevention from 2011 until 2016.

Statistical analysis was performed using IBM SPSS statistics 29.0 for Windows (IBM SPSSINC., Chicago, IL, USA). Because of non-normal distributions of most of our variables, medians and ranges were presented after generating an individual median value per person in case of multiple measurements, and comparisons between groups were performed with the Mann–Whitney U test. We applied linear mixed model analysis to assess changes in plasma copper and zinc levels over time.

## Figures and Tables

**Table 1:** Copper and zinc levels (in µmol/L) in children with classic A-T.

(**a**) **Classic A-T, age 1–17 years**				
		**A-T** **(*n* = 78 samples from 24 children)**	**Control** **(*n* = 179 samples/children)** **Al Fify et al. [14]**	***p* value**
Copper in µmol/L	Median	18.4	17.2	0.297
	Range	11.5–34.3	11.7–25.8	
Zinc in µmol/L	Median	11.2	11.9	**0.005**
	Range	7.6–14.0	8.6–17.0	
Copper/zinc ratio	Median	1.6	1.4	**0.011**
	Range	0.9–4.0	0.8–2.8	
(**b) Classic A-T, age 1–11 years**				
		**A-T** **(*n* = 48 samples from 17 children)**	**Control** **(*n* = 133 samples/children)** **Al Fify et al. [14]**	***p* value**
Copper in µmol/L	Median	19.2	17.7	0.116
	Range	12.9–34.3	11.7–25.8	
Zinc in µmol/L	Median	11.0	11.9	**<0.001**
	Range	7.6–12.8	8.6–15.9	
Copper/zinc ratio	Median	1.8	1.4	**0.003**
	Range	1.1–4.0	0.9–2.8	
(**c**) **Classic A-T, age 12–17 years**				
		**A-T** **(*n* = 30 samples from 10 children)**	**Control** **(*n* = 46 samples/children)** **Al Fify et al. [14]**	***p* value**
Copper in µmol/L	Median	16.6	15.7	0.797
	Range	10.9–19.4	11.7–22.1	
Zinc in µmol/L	Median	12.7	11.9	0.932
	Range	9.0–14.0	9.6–17.0	
Copper/zinc ratio	Median	1.4	1.3	0.535
	Range	0.9–1.8	0.8–1.8	

**Table 2:** Copper and zinc levels (in µmol/L) in adults with A-T.

(**a**) **Adults with classic A-T**				
		**Classic A-T (*n* = 29 samples from 8 adults)**	**Control (*n* = 4342 samples/adults)**	***p* value**
Copper in µmol/L	Median	14.8	18.0	**0.003**
	Range	12.1–18.1	5.0–46.7	
Zinc in µmol/L	Median	12.0	12.4	0.592
	Range	9.7–14.3	6.7–30.4	
Copper/zinc ratio	Median	1.3	1.5	0.076
	Range	0.8–1.6	0.4–6.3	
(**b**) **Adults with variant A-T**				
		**Variant A-T (*n* = 20 samples from 6 adults)**	**Control (*n* = 4342 samples/adults)**	***p* value**
Copper in µmol/L	Median	18.0	18.0	0.739
	Range	17.0–21.8	5.0–46.7	
Zinc in µmol/L	Median	10.7	12.4	0.088
	Range	9.6–14.1	6.7–30.4	
Copper/zinc ratio	Median	1.7	1.5	0.178
	Range	1.2–2.2	0.4–6.3	

## Data Availability

The data that support the findings of this study are available from the corresponding author upon reasonable request.

## Abbreviations

The following abbreviations are used in this manuscript:

A-T	Ataxia–Telangiectasia
ATM	Ataxia–Telangiectasia Mutated
